# Whole-Genome Analysis of Domestic Chicken Selection Lines Suggests Segregating Variation in ERV Makeups

**DOI:** 10.3390/genes10020162

**Published:** 2019-02-20

**Authors:** Mats E. Pettersson, Patric Jern

**Affiliations:** Science for Life Laboratory, Department of Medical Biochemistry and Microbiology, Uppsala University, Box 582, SE-75123 Uppsala, Sweden; Mats.Pettersson@imbim.uu.se

**Keywords:** endogenous retrovirus, host genome, evolution, segregation

## Abstract

Retroviruses have invaded vertebrate hosts for millions of years and left an extensive endogenous retrovirus (ERV) record in the host genomes, which provides a remarkable source for an evolutionary perspective on retrovirus-host associations. Here we identified ERV variation across whole-genomes from two chicken lines, derived from a common founder population subjected to 50 years of bi-directional selection on body weight, and a distantly related domestic chicken line as a comparison outgroup. Candidate ERV loci, where at least one of the chicken lines indicated distinct differences, were analyzed for adjacent host genomic landscapes, selective sweeps, and compared by sequence associations to reference assembly ERVs in phylogenetic analyses. Current data does not support selection acting on specific ERV loci in the domestic chicken lines, as determined by presence inside selective sweeps or composition of adjacent host genes. The varying ERV records among the domestic chicken lines associated broadly across the assembly ERV phylogeny, indicating that the observed insertion differences result from pre-existing and segregating ERV loci in the host populations. Thus, data suggest that the observed differences between the host lineages are best explained by substantial standing ERV variation within host populations, and indicates that even truncated, presumably old, ERVs have not yet become fixed in the host population.

## 1. Introduction

Retroviruses have infiltrated vertebrate germline for millions of years by integrating as proviruses in host DNA, which have then passed down to the offspring through generations as inherited endogenous retroviruses (ERVs). The genomic ERV record represents retroviruses that were replicating at the time of integration and constitutes large fractions of contemporary vertebrate genomes, for example about 7–8% of human DNA [1,2] and about 3% of the chicken genome [3]. The genomic ERV record thus presents a remarkable source for an evolutionary perspective on the biology and interactions among retroviruses and their hosts.

Diverse sets of ERVs can be identified across all studied vertebrate genome assemblies [4] by screening for structural hallmarks including long terminal repeats (LTRs), which flank the ERV *gag, pol*, and *env* genes [5]. Over time, ERV loci may become fixed in the host population, either due to genetic drift of those loci that are least harmful or due to selection on beneficial insertions [2]. ERV contributions to the host genome structure and function include providing a substrate for genomic recombination, and effects on the host transcriptome resulting from their integration and expression with diverse effects on host genome function and evolution. Among positive effects are the expression of viral gene products as useful new genes in the host [6], modification of chromosomal gene expression by ERVs including promoter, enhancer, and insulator functions, as well as alternative splice signals from ERV integrations in host transcription units or adjacent to chromosomal genes [2,7]. On the other hand, is the potential for host gene disruption, as well as the potential for somatic spread of replicating retroviruses leading to pathogenic consequences for the host [2,8,9]. ERV-mediated genomic recombination can further contribute to the organization and plasticity of the host’s genome [10,11,12,13]. Overall, it is plausible that ERVs have had considerable effects on host genome function and evolution across the entire vertebrate lineage, by shuffling genomic regions, exons, and regulatory genetic sequences into new contexts and thereby altering the dynamic functions of the host DNA.

It is desirable to identify orthologous ERV loci across the compared host lineages in order to evaluate potential effects of retroviruses and ERVs on host biology because it allows for connecting ERV integrations to host phenotypic differences and evolutionary history. ERV studies have benefited from recent advancements in sequencing technology and a growing catalogue of reference host genome assemblies, where much focus has been placed on comparing ERV records across related host species reference genome assemblies, an approach that suffers from undersampling of the diversity within vertebrate species, and thus presents challenges for reaching a better understanding of potential factors that contribute to the long-term retrovirus-host associations [4]. More recently, studies utilizing re-sequencing data to target searches for integration differences among selected ERVs within a host population to explain activities during recent evolution have made efforts to address this issue for specific virus types in host populations [14,15].

In an attempt to further explore ERV-host associations in a hitherto un-examined system, we make use of an artificial selection system where selection lines of domestic chicken that have been undergoing strong bi-directional selection on body weight at eight weeks of age for more than 50 years [16]. This selection-scheme, from a single founder population, has generated extreme phenotypes with more than 10-fold difference in average weights between the two chicken lines. We utilized whole-genome re-sequencing data from these chicken lines, as well as an outgroup commercial chicken line, to investigate ERV insertion variation and potential evolutionary contributions from inherited ERVs on host genome function.

Domestic animals provide rare possibilities, currently not feasible in human biomedicine, to study connections between genes, phenotypes, and biological function [17]. Crossbreeding of domestic animals is also a useful tool to determine genomic differences, making it possible to apply genetic analyses to reveal loci controlling phenotypic traits that have been selected during domestication [18].

The rationale for utilizing chicken as a model dates back more than 100 years to pioneering studies of retroviruses and ERVs, reviewed in [19,20], and the availability of sequence data from the chicken selection pedigree established in 1957 (see above), which, measured by the response in phenotypic traits and single nucleotide polymorphism (SNP) allele-frequency divergence, has accumulated changes that have been estimated by Johansson et al. to require about 5000 years to evolve in natural populations [21]. Overall, the chicken selection lines present a promising model for identifying ERV divergence and interpreting observations in the context of previously known results in this system, thereby estimating ERV contributions to dynamics of complex genetic traits of their hosts.

Here, we identify ERV insertion differences across available re-sequenced genomes derived from the two bi-directionally growth-selected chicken lines and compare candidate ERV loci with a commercial layer chicken outgroup. We map insertions and deletions to establish their positions relative to the adjacent host genomic landscape and compare candidate loci associated by sequence similarity to ERVs identified in the Red junglefowl reference assembly (version galGal3) along with reference retroviral sequences within a phylogenetic framework.

## 2. Materials and Methods

### 2.1. Domestic Chicken Selection Lines

Whole-genome re-sequenced DNA from domestic chicken selection lines was analyzed for differences in ERV makeups as potential markers for effects of ERVs on host genome function and evolution, see Table 1. For this purpose, we utilized a well-studied model system established in 1957, where two growth-selected lines of chicken were developed from a single founder population by bi-directional selection of body weight at 56 days of age for more than 40 generations and kept as closed populations [16]. The average body weights of individuals from respective selection lines (H: high-growth; L: low-growth) differ more than 10-fold today, and, in addition to the response to the selection of body weight, the lines have accumulated significant differences in feeding behavior and food consumption [22]. The chicken selection lines (H and L) were analyzed together with the commercial white leghorn chicken (W) for whole-genome comparisons as previously described in a study of domestication sweeps [18]. Mate-pair sequence read libraries, with read length 50 nt times two and approximately 3 to 4 kb insert sizes were produced using high throughput SOLiD sequencing technology, see Table 1, and mapped to the Red junglefowl reference genome assembly (version galGal3, accessed from the UCSC Genome Browser, http://genome.ucsc.edu) by Rubin et al. [18].

### 2.2. Endogenous Retrovirus Mapping

Briefly, to allow identification of reference as well as non-reference assembly ERVs using mate-pair short reads sequencing technology, we applied a strategy where reads were mapped to an independent ERV library and then located along host chromosomes by anchoring their mate-pair reads to positions in the flanking host DNA. The RetroTector software [5] was used to mine the Red junglefowl reference genome assembly (version galGal3) for ERV sequences to construct an independent reference library for mapping ERV-associated SOLiD sequencing short reads for each (H, L, and W, see above) chicken selection line [18] using the SHRiMP2 software [23]. To identify ERV-host DNA junctions, SHRiMP2 ERV-associated reads scoring ≥400 were paired with reads that target unique chromosomal flanking sequences [18]. The number of expected loci including full-length ERVs, truncated ERVs, and solo-LTRs [24], which are the results from homologous recombination between the two provirus LTRs, can be estimated to be about 20 times more frequent than the number of full-length ERVs based on previous evaluations [1], which serves as a conservative starting point for analyses. As our RetroTector analyses of the galGal3 reference assembly identified 532 high-quality ERVs scoring ≥300 (as previously discussed [5]), a putative target of around 10,000 candidate ERV loci was used to determine conditions for reads clustering at ERV-host DNA junctions. Conservative (top) scores were used for chromosomal DNA positions mapped by Rubin et al. [18] and SHRiMP2 ERV-associated read scores (ranging 400–493) were used at ≥425. The up- and downstream ERV-host DNA junctions were clustered separately considering mate-pair reads insert size of about 3500 nt, which reflects the maximum chromosomal flanking distance to the ERV integration, and requiring short reads to cover at least 2% of that length. Up- and downstream ERV-host DNA junctions were paired given shared orientation, read associations to target reference ERV sequences, indications of both 5′- and 3′-ERV flanking sequences, and separation by less than about 20 kb to accommodate expected ERV lengths of around 7–11 kb and potential secondary transposable element integrations into the candidate ERV. Together, these clustering conditions indicated 12,709 candidate ERV loci and additional ERV-associated reads with relaxed mapping scores (≥400) were appended to the identified ERV-host DNA junctions.

### 2.3. Endogenous Retrovirus Integration Variation

Candidate ERV loci were tested for read mapping differences across the re-sequenced chicken lines (H, L, and W, see above) using Fisher’s exact test if the minimum observed read counts at the locus were fewer than 15 and otherwise by comparison to the Chi distribution. Loci where short reads were missing in one or two of the three chicken lines were kept for further analyses if p-values for ERV-associated read counts passed the conservative threshold (*p* < 4 × 10^−6^) after Bonferroni correction for multiple testing.

### 2.4. Endogenous Retrovirus Integration Landscape

The Red junglefowl (galGal3) reference gene dataset was downloaded from the UCSC genome browser (http://genome.ucsc.edu) and intersected with positions for candidate ERV loci in order to explore biological significance of genes located adjacent to ERVs and their potential associations with the chicken selection line phenotypes. Associations among chromosomal genes and ERVs, intragenic as well as intergenic positions covering 150 kb up- and downstream of reference gene transcription start sites were analyzed. Candidate ERV loci were intersected with sweep regions previously determined for the H and L chicken selection lines [18,21]. Chromosomal reference genes identified adjacent to ERV loci were included in searches at the database for annotation, visualization, and integrated discovery (DAVID at https://david.ncifcrf.gov/) to explore biological impact of differences in ERV integrations across the H and L chicken selection lines.

### 2.5. Phylogenetic Framework

Phylogenetic analyses of ERVs identified in the reference assembly (version galGal3) together with reference retrovirus sequences were performed as previously described [4,25,26]. Briefly, high-quality ERVs (RetroTector score ≥ 300) identified in the reference chicken assembly (galGal3) were split according to conserved motifs and phylogenetically informative segments across the ERV *gag* and *pol* genes for multiple sequence alignments that were concatenated for phylogenetic analysis using FastTree2 [27]. The resulting phylogenetic tree was rooted using the *Caenorhabditis elegans* retrotransposon Cer1 (GenBank accession no. U15406), and visualized using FigTree v1.4.2 (http://tree.bio.ed.ac.uk/software/figtree/).

## 3. Results

Whole-genomes from domestic High-Growth (H), Low-Growth (L), and White Leghorn (W) chicken selection lines were previously sequenced using high throughput SOLiD technology and mapped to the chicken reference assembly (version galGal3) by Rubin et al. [18], see Table 1. Here, we utilized the RetroTector software [5] to identify ERVs in the Red junglefowl (version galGal3) assembly, which were used as an independent sequence library to map ERV-associated reads from the re-sequenced chicken lines that could then be mated with their respective chromosomal mapping reads for locating ERV-host DNA insertion junctions, even in cases where the insertion was absent from the reference assembly. Up- and downstream ERV-host junctions were clustered and paired using stringency criteria tuned for identifying about 10,000 loci, the expected number of ERV and solo LTR loci based on RetroTector results and a previously estimated 1:20 ratio between complete ERVs and solo LTRs, which are generated by homologous recombination between the two proviral LTRs [1]. The clustering of paired sequence reads identified 12,709 candidate ERV loci, of which 8340 candidate loci indicated distinct differences, measured as absence or near-absence of ERV-associated reads in at least one of the three compared chicken lines. Bonferroni correction for multiple testing left 369 differentiated candidate ERV loci. Among these candidate loci, 115 ERVs were adjacent to, or located within, 229 host genes considering 150 kb distances up- and downstream of the candidate loci, see Table 2 and Supplementary Information Table S1.

**Figure 1:**
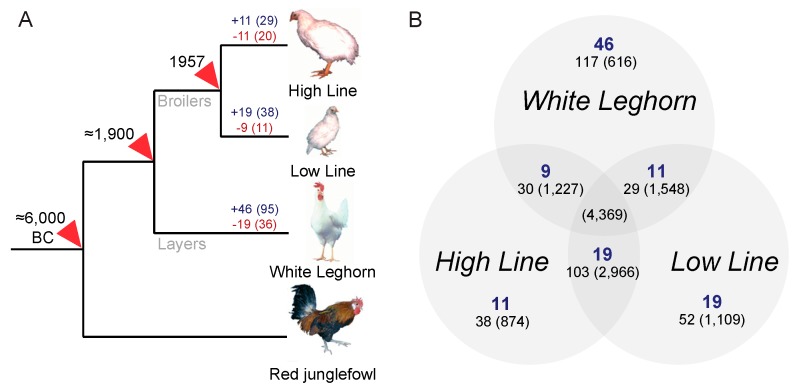
Chicken selection lines and ERV variation. (**A**) Phylogeny of analyzed fowl modified from Rubin et al. [18]. Blue numbers above branches indicate candidate branch-specific ERV insertions and red numbers indicate candidate branch-specific missing ERVs at the analyzed loci, see Table 2. Numbers in brackets indicate ERV-associated host genes. (**B**) Venn diagram showing distribution of identified candidate ERV differences across the domestic chicken selection lines. Blue numbers indicate counts of ERVs only found in the respective chicken line adjacent to genes. The numbers below represent the number of candidate loci after correction for multiple testing, see Table 2, and numbers within brackets show the corresponding number of candidate loci before correction.

The bi-directionally growth-selected chicken lines (H and L) diverged from a single broiler founder population about 60 years ago and were separated more than 100 years ago from the branch leading to the comparison outgroup represented here by the commercial White Leghorn (layer) chicken. For reference, the compared chicken lines share a relatively recent common ancestry, compared to the reference genome assembly, generated from the Red junglefowl, *Gallus gallus*, which was separated from the investigated chicken lines about 8000 years ago when chicken was first domesticated, see Figure 1. However, even this split is recent compared to datasets that have been the subject of previous studies [4,14,15,26], and thus the use of ERV loci comparison in a small host pedigree, such as the domestic chicken lines, relies on that integration differences may be observed as a result from selection during domestication that could require many thousands of years to become fixed in wild host populations [21]. The observed branch-specific ERV loci differences across the domestic chicken selection lines broadly reflects the time scale after divergence as the growth-selected broiler chicken lines (H and L) were separated about half of the time since the layer outgroup (W) separated from the domesticated broiler chickens, see Figure 1, indicating that differences in ERV makeups may provide potential traceable markers for host evolution, see Figure 1, Table 2.

**Figure 2:**
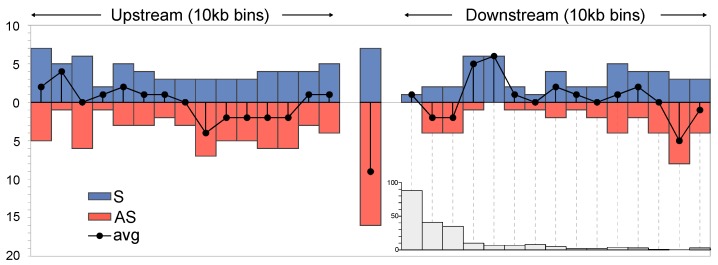
Chicken ERV integration landscape relative host genes (upstream, intragenic, and downstream) and orientation. Blue and red histograms show the number of identified host genes at various distances to the ERV in sense (blue) and antisense (red) relative orientations, and average points are indicated for each bin. The grey histogram insert indicates the number of analyzed genes at downstream distances, split into 10 kb bins, with respect to nearby ERVs.

To explore potential connections between the observed divergent ERV loci across the domestic chicken lines, we intersected chromosomal positions with the reference assembly host genes (version galGal3, downloaded from the UCSC genome browser, http://genome.ucsc.edu) and previously determined selective sweeps for the H and L chicken selection lines [18,21]. Although some ERVs overlapped with domestication sweep regions, see Supplementary Information Table S1, the observed overlap did not deviate significantly from the expectation (*p* = 0.1, binomial test), given the size of the sweep areas and the number of detected ERV insertions elsewhere in the host genomes. In addition, gene ontology searches were inconclusive and could not establish links between ERVs and adjacent host genes that could help explain the distinct phenotypes. We, therefore, analyzed candidate ERV insertion orientations and distances relative to host genes. Candidate ERV loci within host gene transcripts show a clear bias in antisense orientation relative to the host gene transcript, which could be explained by purifying selection due to potential splice interference from canonical splice signals as previously discussed [28]. Intergenic ERV orientations relative to host genes fluctuate up- and downstream and a bias pattern is not clear given the limited data, see Figure 2. It thus appears that intergenic ERV insertions may not influence host genome function to the same extent as intragenic ERVs.

**Figure 3:**
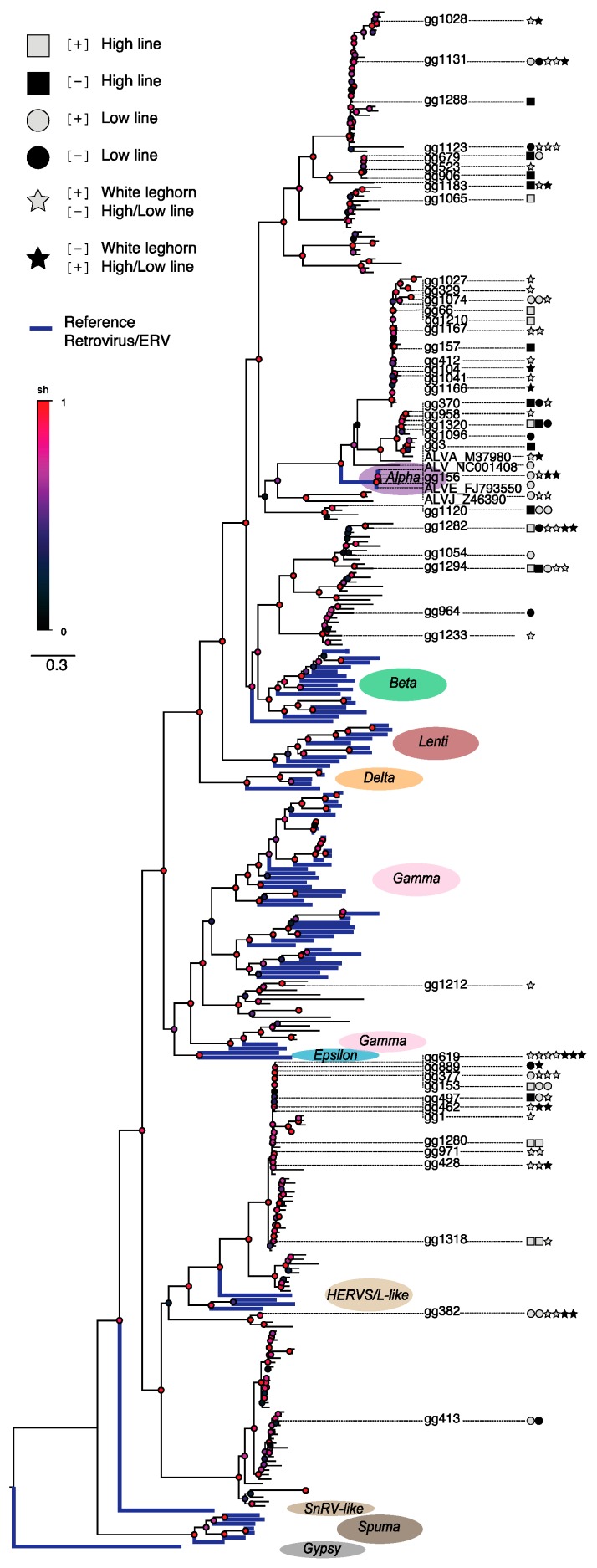
Chicken ERV phylogenetic tree based on Gag and Pol motifs as previously described [4,25]. Thick blue lines indicate reference retroviral and ERV sequences. Candidate ERV-associated loci differences between the H, L, and W chicken are indicated by symbols next to the ERV IDs referring to the RetroTector analysis of the chicken (galGal3) assembly (see FASTA sequences and loci annotations in Supplementary Information Data S1). The complete phylogenetic tree is presented in nexus file format (Supplementary Information Data S2) for rendering in a tree drawing software such as FigTree.

To investigate relationships between the observed ERV loci varying across the analyzed chicken lines, we constructed a phylogenetic tree based on ERVs identified by the RetroTector software [5] in the Red junglefowl reference assembly (version galGal3) and appended reference retroviral sequences for comparisons as previously described [4,25,26]. Since the insert sizes and read lengths of ERV-associated mate-pair reads only allow limited coverage into the candidate ERV loci, it is useful to align reads to reference assembly ERVs that could build a phylogenetic framework, and from which the best ERV match for candidate ERV loci by can be determined, see Figure 3.

In agreement with the observed lack of significant associations between candidate ERV loci and adjacent host genes (see above), divergent ERV loci in the domestic H, L, and W chicken lines located across the phylogenetic tree that was rooted on a distant outgroup, rather than being found inside any specific retroviral clade, which is what could be expected if variation was due to retroviral expansion after the last common ancestor. Instead, the result indicates that the observed candidate ERV insertion differences do not result from recent retrovirus replication and integrations as ERVs in one or two of the chicken lineages, but rather it is consistent with standing variation of segregating ERV loci present at the onset of the bi-directional selection experiment as well as during breed formation since the domestication of chicken. Multiple radiations involving candidate ERV loci associated with assembly ERVs showing short terminal branch lengths indicate relatively recent expansions occurring within several retroviral genera across the phylogeny. It seems plausible that these radiations have generated a substantial number of segregating ERV insertions in the domestic chicken lines, thus providing the standing variation that explains the observed differences in ERV makeups, and that the number of divergent ERV loci is largely a product of the accelerated genomic divergence caused by the strong selection imposed on the H and L lines specifically, as well as directed selection of host features during domestication, which has affected all three studied (H, L, and W) chicken lines.

## 4. Discussion

The known breeding history and well-studied phenotypic traits among domestic animals make them first-rate model organisms to identify potential ERV contributions to biological functions and dynamics of complex genetic traits. Rare genomic changes resulting in host phenotypes that would require thousands of years to establish, or become lost, in wild host populations may be selected for in domestic settings during fewer generations [17,21,29]. This type of genomic data presents an excellent chance to study differences in genomic ERV makeups across many chicken selection lines.

Here, we utilized whole-genome sequences from two bi-directionally growth-selected domestic broiler chicken lines and a distantly related domestic layer chicken line [18] for identifying and comparing candidate ERV insertion differences. Using an independent ERV search library, it is possible to identify non-reference assembly ERV in the different host lineages. We show that the domestic chicken carries a large number of segregating ERVs, evidenced by the observation that 65% of detected loci display a nominal difference in frequencies and more than 350 insertions are significantly differentiated. Standing variation has previously been shown to contribute to the majority of the alleles under selection in the H and L lines [21,22,29] and here we show that segregating ERVs are a part of this variation, and thus they form part of the potential substrate for selection in these, and other, chicken lines.

As high-throughput parallel sequencing technologies generate short reads and limited coverage into the ERV loci depending on mate-pair insert sizes for reliable chromosomal anchoring, we utilized ERVs identified in the Red junglefowl reference assembly to generate an independent ERV search library and anchored loci to chromosomal positions by ERV-associated short reads mate-pair mapping. Despite limited ERV sequence coverage, it is thus possible to associate the best fit for candidate ERV loci reads with assembly ERV sequences, which could be used to construct phylogenetic frameworks and to determine associations between ERV loci and host genomic landscapes.

The whole-genome sequences were generated from pooled individual DNA, see Table 1, which complicates assessments of ERV presence/absence and we, therefore, used a conservative approach by considering loci where one or two of the three domestic chicken lines indicated missing or present ERV-associated reads. Given the large phenotypic differences between the high- and low-growth chicken lines, the domestic animal model presents a promising system to determine potential influences from ERVs on host genome structure and function.

However, although gene ontology searches for genes adjacent to divergent candidate ERV loci could not explain host phenotypic variation, which could be due to the known highly polygenic nature of the trait under selection [21,29], intersection of ERV loci positions with previously determined domestication sweep signals [18,21,29] showed only a weak association. Together, the results suggest that pre-existing ERV variation derived from a common host ancestor segregate in the domestic chicken selection lines today. This notion is supported by the estimated age range of loci that are divergent loci between the domestic chicken lines, which include loci that are presumably old, based on sequence similarities to re-constructed reference assembly ERVs, as well as newer reference assembly ERV insertions, indicating that the divergence represents a frequency shift among ERV loci that segregate in the host population.

Similar observations have also recently been made in other vertebrate host populations [26], demonstrating limitations when assessing historic retrovirus activities from the genomic ERV record using reference assemblies compared to host population data [30], due to the severe sampling effect and associated loss of diversity introduced by reducing a species population down to a single reference genome.

While it can be informative to study ERV variation from host species assemblies covering multiple species over long evolutionary time scales [4,25], analyses along single species phylogenies provide additional information regarding ERV variation and expression [31]. To analyze more recent ERV activities, it has also been successful to employ targeted analysis of specific ERVs in single host species population data [14,15]. However, sampling constraints complicate identification of standing variations in ERV makeups in these systems, and the broad searches in controlled genome groups that the domestic animal selection pedigrees provide are not easily achieved under such conditions. By narrowing the time scale using domestic and wild animal pedigrees, it has been possible to estimate segregating ERV variation for a broad range of ERV clades in host populations [26]. Use of PCR to investigate polymorphisms and incomplete lineage sorting was recently demonstrated for young ERVs [32], and further refining these types of studies by analyzing the recently diverged growth-selected chicken pedigree in this study, we conclude that standing ERV variation is a common feature in contemporary vertebrate populations.

In summary, it appears increasingly important to employ careful experimental design to control the occurrence of artifacts and incorrect inferences due to unbalanced sampling in analyses aimed at evaluating host species ERV makeups. In order to obtain valid comparisons from population and distantly related genomes, it is valuable to focus on well-known pedigrees like those offered by domestic animal selection lines, where the prior knowledge makes it possible to compare observed patterns with expectations that are based on the evolutionary context of the specific case with higher precision than is generally achievable in natural populations. Sequencing and analyses of domestic animal populations and single genomes from known selection pedigrees facilitated by improved sequencing technologies that provide depth and coverage over long insertion sizes together with newly developed and fine-tuned analysis methods will facilitate mapping of ERVs previously not feasible and thereby generate new knowledge about contributions from retroviruses and ERVs to host genome function and evolution.

## Figures and Tables

**Table 1:** Re-sequenced chicken selection lines and endogenous retrovirus (ERV) associated reads.

Pooled Genomes ^1^	Short Name	Library ^2^	n ^3^	Coverage ^4^	ERV Assoc. Reads	ERV-Host Read Pairs
High-growth line	H	ugc_208	11	5.53x	233,621	82,719
Low-growth line	L	ugc_209	11	5.19x	239,189	98,942
White leghorn	W	ugc_254	11	3.37x	191,873	90,759

^1^ High throughput SOLiD sequencing mate-pair libraries as previously described [18]. White leghorn chicken was used as an outgroup in comparisons. ^2^ Approximately 3500 nt mate-pair library gap lengths mapped to the chicken genome assembly (version galGal3) [18]. ^3^ Numbers of pooled individuals in SOLiD sequencing. ^4^ Sequencing coverage as previously described [18].

**Table 2:** Candidate ERV loci.

Chicken ^1^	ERV Candidate Loci	ERV Loci (Corrected) ^2^	ERVs Adjacent to Genes ^3^	Genes Adjacent to ERVs ^3^
H••	874	38	11	29
•L•	1109	52	19	38
••W	616	117	46	95
HL•	2966	103	19	36
H•W	1227	30	9	11
•LW	1548	29	11	20
HLW	4369	nd	nd	nd

^1^ Re-sequenced chicken selection lines selection lines: H (High-Growth line), L (Low-Growth line) and W (White Leghorn). ^2^ Candidate ERV loci after Bonferroni correction (*p* < 4 × 10^−6^) where ERV-associated reads indicate distinct differences (present/missing reads) in one of the chicken lines compared to the others. ^3^ Within 150 kb.

## Supplementary Materials

The following are available online at https://www.mdpi.com/2073-4425/10/2/162/s1, Data S1: galGalERV FASTA sequences, Data S2: galGalERV phylogeny in nexus format, Table S1: galGalERV loci variation and genomic landscape.
