# Supplementary material for: Whole-Genome Analysis of Domestic Chicken Selection Lines Suggests Segregating Variation in ERV Makeups
Source: Genes (Basel). 2019 Feb 20;10(2):162. doi: 10.3390/genes10020162 (PMC6410134; doi:10.3390/genes10020162)
Supplement: Supplementary file 1 [file genes-10-00162-s001.pdf]

Table S1.

ERV Locus(Ori)	reads. H	reads L	reads W	P-value	Chicken	ERV Ref	Chr	Site	refGene	txStart	txEnd	ERV txUp	ERV txIn	ERV txDown	Sweep
**c1.1(+)**	131	136	0	1.60E-30	HL•	ALVA	1	11428668	HGF	11443008	11513810	S	•	•	NO
**c1.2(+)**	25	32	0	1.66E-06	HL•	gg462	1	51556180	TOB2	51523188	51530048	•	•	S	NO
**c1.2(+)**	25	32	0	1.66E-06	HL•	gg462	1	51556180	RANGAP1	51620376	51639709	S	•	•	NO
**c1.2(+)**	25	32	0	1.66E-06	HL•	gg462	1	51556180	XRCC6	51450748	51436174	AS	•	•	NO
**c1.2(+)**	25	32	0	1.66E-06	HL•	gg462	1	51556180	ACO2	51512148	51491243	AS	•	•	NO
**c1.2(+)**	25	32	0	1.66E-06	HL•	gg462	1	51556180	TEF	51563882	51548176	•	AS	•	NO
**c1.2(+)**	25	32	0	1.66E-06	HL•	gg462	1	51556180	L3MBTL2	51659859	51645263	•	•	AS	NO
**c1.3(+)**	2	0	77	3.58E-19	H•W	gg1123	1	54821503	LARGE	54793760	54990691	•	S	•	NO
**c1.4(+)**	13	0	86	1.16E-17	H•W	gg1282	1	64586799	AKR1B10	64618852	64610741	•	•	AS	NO
**c1.5(+)**	0	27	61	8.43E-11	•LW	gg678	1	70395382	ARNTL2	70297388	70333505	•	•	S	NO
**c1.5(+)**	0	27	61	8.43E-11	•LW	gg678	1	70395382	PPFIBP1	70419133	70459954	S	•	•	NO
**c1.6(+)**	0	1	74	7.22E-19	•LW	gg9	1	76417209	NTF3	76307753	76308649	•	•	S	NO
**c1.7(+)**	0	0	60	3.65E-16	••W	gg1318	1	116485436	OTC	116487231	116461326	•	AS	•	NO
**c1.8(+)**	81	101	0	8.07E-21	HL•	gg1028	1	130808531	NLGN4X	130904687	131025946	S	•	•	NO
**c1.9(+)**	31	0	0	3.44E-09	H••	gg1065	1	143060091	COL4A2	143165550	143306843	S	•	•	YES
**c1.9(+)**	31	0	0	3.44E-09	H••	gg1065	1	143060091	COL4A1	143165166	143054857	•	AS	•	YES
**c1.10(+)**	0	0	32	9.02E-09	••W	gg1131	1	150564881	DCT	150688696	150710209	S	•	•	NO
**c1.11(+)**	0	0	148	2.57E-40	••W	gg1041	1	150732360	DCT	150688696	150710209	•	•	S	NO
**c1.12(+)**	0	102	0	8.43E-26	•L•	ALVJ	1	156989892	SPRY2	156881807	156890285	•	•	S	NO
**c1.13(+)**	4	0	38	2.76E-08	H•W	gg889	1	189946644	MRE11A	189912121	189930815	•	•	S	NO
**c1.13(+)**	4	0	38	2.76E-08	H•W	gg889	1	189946644	FUT4	189894356	189893301	AS	•	•	NO
**c1.13(+)**	4	0	38	2.76E-08	H•W	gg889	1	189946644	MED17	190082536	190068502	•	•	AS	NO
**c1.14(+)**	0	0	102	1.48E-27	••W	gg619	1	189961050	MRE11A	189912121	189930815	•	•	S	NO
**c1.14(+)**	0	0	102	1.48E-27	••W	gg619	1	189961050	FUT4	189894356	189893301	AS	•	•	NO
**c1.14(+)**	0	0	102	1.48E-27	••W	gg619	1	189961050	MED17	190082536	190068502	•	•	AS	NO

ERV Locus(Ori)	reads. H	reads L	reads W	P-value	Chicken	ERV Ref	Chr	Site	refGene	txStart	txEnd	ERV txUp	ERV txIn	ERV txDown	Sweep
**c1.15(+)**	190	192	0	1.16E-43	HL•	gg156	1	192139230	NOX4	192020463	192113365	•	•	S	NO
**c1.15(+)**	190	192	0	1.16E-43	HL•	gg156	1	192139230	GRM5	192231216	192474226	S	•	•	NO
**c1.15(+)**	190	192	0	1.16E-43	HL•	gg156	1	192139230	TYR	192188837	192138670	•	AS	•	NO
**c1.16(+)**	60	0	0	2.33E-17	H••	gg1318	1	199469010	HBG1	199433572	199434763	•	•	S	NO
**c1.16(+)**	60	0	0	2.33E-17	H••	gg1318	1	199469010	HBG2	199436905	199441999	•	•	S	NO
**c1.16(+)**	60	0	0	2.33E-17	H••	gg1318	1	199469010	HBE1	199436937	199438083	•	•	S	NO
**c1.16(+)**	60	0	0	2.33E-17	H••	gg1318	1	199469010	HBE	199444834	199446373	•	•	S	NO
**c1.16(+)**	60	0	0	2.33E-17	H••	gg1318	1	199469010	OR51M1	199454178	199455138	•	•	S	NO
**c1.16(+)**	60	0	0	2.33E-17	H••	gg1318	1	199469010	CCKBR	199325913	199323532	AS	•	•	NO
**c1.16(+)**	60	0	0	2.33E-17	H••	gg1318	1	199469010	RPS11	199346749	199344203	AS	•	•	NO
**c1.16(+)**	60	0	0	2.33E-17	H••	gg1318	1	199469010	FOLR1	199405661	199402555	AS	•	•	NO
**c1.16(+)**	60	0	0	2.33E-17	H••	gg1318	1	199469010	OR52R1	199461195	199460248	AS	•	•	NO
**c1.16(+)**	60	0	0	2.33E-17	H••	gg1318	1	199469010	RRM1	199560670	199540616	•	•	AS	NO
**c1.16(+)**	60	0	0	2.33E-17	H••	gg1318	1	199469010	STIM1	199580277	199574923	•	•	AS	NO
**c2.1(+)**	0	0	150	1.16E-40	••W	gg382	2	11703794	KLF6	11718622	11709659	•	•	AS	NO
**c2.2(+)**	15	106	0	7.22E-20	HL•	gg326	2	21625449	MIR466	21671960	21672029	S	•	•	NO
**c2.3(+)**	0	0	115	2.68E-31	••W	gg619	2	31555588	DFNA5	31560405	31534990	•	AS	•	NO
**c2.3(+)**	0	0	115	2.68E-31	••W	gg619	2	31555588	CYCS	31694280	31693797	•	•	AS	NO
**c2.4(+)**	0	0	34	4.44E-09	••W	gg377	2	31559399	DFNA5	31560405	31534990	•	AS	•	NO
**c2.4(+)**	0	0	34	4.44E-09	••W	gg377	2	31559399	CYCS	31694280	31693797	•	•	AS	NO
**c2.5(+)**	0	23	0	1.92E-06	•L•	gg1093	2	48148838	NT5C3	48041580	48053719	•	•	S	NO
**c2.5(+)**	0	23	0	1.92E-06	•L•	gg1093	2	48148838	FKBP9	48074886	48059096	AS	•	•	NO
**c2.6(+)**	16	84	0	3.34E-15	HL•	gg382	2	72991689	ENS-3	72996485	73002095	S	•	•	NO
**c2.7(+)**	0	0	47	1.80E-12	••W	gg1123	2	91638485	INVS	91576378	91490447	AS	•	•	NO
**c2.7(+)**	0	0	47	1.80E-12	••W	gg1123	2	91638485	STX17	91658317	91626577	•	AS	•	NO
**c2.8(+)**	54	0	86	5.41E-18	H•W	gg1096	2	113036225	PCMTD1	112988563	112942519	AS	•	•	YES
**c2.9(+)**	0	0	75	3.25E-20	••W	gg971	2	147263413	PHF20L1	147372809	147420655	S	•	•	NO

ERV Locus(Ori)	reads. H	reads L	reads W	P-value	Chicken	ERV Ref	Chr	Site	refGene	txStart	txEnd	ERV txUp	ERV txIn	ERV txDown	Sweep
**c2.10(+)**	0	11	55	1.98E-10	•LW	gg1306	2	154731703	PUF60	154866396	154841396	•	•	AS	NO
**c3.1(+)**	2	0	28	1.44E-06	H•W	gg1131	3	1026772	FANCL	920584	891593	AS	•	•	YES
**c3.2(+)**	0	0	45	4.03E-12	••W	gg1282	3	7674302	EHD3	7640401	7615214	AS	•	•	NO
**c3.3(+)**	0	0	33	7.09E-09	••W	gg1282	3	15997819	BMP2	16013577	16010636	•	•	AS	NO
**c3.4(+)**	0	54	0	2.15E-13	•L•	gg413	3	53808158	GTF2H5	53785643	53790123	•	•	S	YES
**c3.5(+)**	0	0	32	9.02E-09	••W	gg1212	3	83522828	TMEM30A	83515836	83529332	•	S	•	NO
**c3.5(+)**	0	0	32	9.02E-09	••W	gg1212	3	83522828	COL12A1	83548164	83648677	S	•	•	NO
**c3.6(+)**	0	2	31	3.87E-07	•LW	gg1183	3	101969430	DDX1	101837290	101859293	•	•	S	NO
**c3.6(+)**	0	2	31	3.87E-07	•LW	gg1183	3	101969430	MYCN	102114356	102117496	S	•	•	NO
**c3.7(+)**	0	0	64	3.78E-17	••W	gg1028	3	101970492	DDX1	101837290	101859293	•	•	S	NO
**c3.7(+)**	0	0	64	3.78E-17	••W	gg1028	3	101970492	MYCN	102114356	102117496	S	•	•	NO
**c3.8(+)**	0	38	16	3.25E-06	•LW	gg105	3	102056888	MYCN	102114356	102117496	S	•	•	NO
**c3.9(+)**	0	42	0	9.76E-11	•L•	gg382	3	104077250	OXSR1	103929131	103927497	AS	•	•	NO
**c4.1(+)**	0	44	0	4.29E-11	•L•	gg1120	4	13347841	DCX	13329700	13403236	•	S	•	YES
**c4.2(+)**	0	40	41	1.75E-08	•LW	gg497	4	16438189	LAMP2	16551748	16568477	S	•	•	NO
**c4.2(+)**	0	40	41	1.75E-08	•LW	gg497	4	16438189	NKAP	16552522	16619063	S	•	•	NO
**c4.2(+)**	0	40	41	1.75E-08	•LW	gg497	4	16438189	MCTS1	16521219	16513179	•	•	AS	NO
**c4.2(+)**	0	40	41	1.75E-08	•LW	gg497	4	16438189	ATP1B4	16577357	16570194	•	•	AS	NO
**c4.3(+)**	32	0	10	1.25E-06	H•W	gg964	4	45139024	FBXO8	45153740	45136922	•	AS	•	NO
**c4.4(+)**	0	0	75	3.25E-20	••W	gg377	4	52979760	CENPC1	53115873	53083976	•	•	AS	NO
**c4.5(+)**	0	34	0	1.10E-08	•L•	gg1245	4	75483212	CCKAR	75629863	75636710	S	•	•	NO
**c4.6(+)**	0	0	194	1.61E-52	••W	gg448	4	82247953	EVC	82118410	82170324	•	•	S	NO
**c4.6(+)**	0	0	194	1.61E-52	••W	gg448	4	82247953	EVC2	82118333	82054939	AS	•	•	NO
**c4.6(+)**	0	0	194	1.61E-52	••W	gg448	4	82247953	CRMP1	82218120	82175811	AS	•	•	NO
**c5.1(+)**	40	0	16	2.97E-08	H•W	gg105	5	4425542	KCNA4	4540765	4536092	•	•	AS	NO
**c5.2(+)**	28	26	0	2.41E-06	HL•	gg619	5	30292874	ERH	30312546	30319567	S	•	•	NO
**c5.2(+)**	28	26	0	2.41E-06	HL•	gg619	5	30292874	SFRS5	30168433	30163795	AS	•	•	NO

ERV Locus(Ori)	reads. H	reads L	reads W	P-value	Chicken	ERV Ref	Chr	Site	refGene	txStart	txEnd	ERV txUp	ERV txIn	ERV txDown	Sweep
**c5.2(+)**	28	26	0	2.41E-06	HL•	gg619	5	30292874	SLC39A9	30312305	30295075	•	•	AS	NO
**c5.3(+)**	9	40	0	3.54E-07	HL•	gg1282	5	50901121	EVL	50903042	50969097	S	•	•	NO
**c5.3(+)**	9	40	0	3.54E-07	HL•	gg1282	5	50901121	YY1	50999597	51022607	S	•	•	NO
**c6.1(+)**	0	32	29	1.92E-06	•LW	gg370	6	27153415	ADD3	27211688	27268533	S	•	•	NO
**c6.1(+)**	0	32	29	1.92E-06	•LW	gg370	6	27153415	MXI1	27285985	27328963	S	•	•	NO
**c7.1(+)**	0	31	0	2.76E-08	•L•	ALVE	7	15952380	CWC22	15874910	15902827	•	•	S	NO
**c7.2(+)**	66	0	0	5.62E-19	H••	gg1093	7	26370332	CNTNAP5	26227278	25970551	AS	•	•	NO
**c7.3(+)**	48	0	0	1.65E-13	H••	gg153	7	37260504	KCNJ3	37363205	37392741	S	•	•	NO
**c7.3(+)**	48	0	0	1.65E-13	H••	gg153	7	37260504	PRPF40A	37195437	37178608	AS	•	•	NO
**c8.1(+)**	0	2	41	8.69E-10	•LW	gg1120	8	1963178	MIR181A-1	2001560	2001664	S	•	•	NO
**c8.1(+)**	0	2	41	8.69E-10	•LW	gg1120	8	1963178	MIR181B-1	2001749	2001838	S	•	•	NO
**c8.1(+)**	0	2	41	8.69E-10	•LW	gg1120	8	1963178	PTPRC	2092242	2034797	•	•	AS	NO
**c8.2(+)**	87	0	0	2.72E-25	H••	gg1280	8	25996006	DIO1	25857745	25862569	•	•	S	NO
**c8.2(+)**	87	0	0	2.72E-25	H••	gg1280	8	25996006	MRPL37	25916521	25919946	•	•	S	NO
**c8.2(+)**	87	0	0	2.72E-25	H••	gg1280	8	25996006	TMEM59	25889363	25881135	AS	•	•	NO
**c8.2(+)**	87	0	0	2.72E-25	H••	gg1280	8	25996006	SSBP3	25974662	25924362	AS	•	•	NO
**c8.2(+)**	87	0	0	2.72E-25	H••	gg1280	8	25996006	DHCR24	26019531	26011324	•	•	AS	NO
**c8.3(+)**	0	0	155	7.82E-42	••W	gg619	8	29604663	GADD45A	29485017	29485191	•	•	S	NO
**c8.3(+)**	0	0	155	7.82E-42	••W	gg619	8	29604663	LOC429115	29563270	29574623	•	•	S	NO
**c8.3(+)**	0	0	155	7.82E-42	••W	gg619	8	29604663	SERBP1	29474561	29461804	AS	•	•	NO
**c8.3(+)**	0	0	155	7.82E-42	••W	gg619	8	29604663	GPR177	29543121	29518976	AS	•	•	NO
**c8.3(+)**	0	0	155	7.82E-42	••W	gg619	8	29604663	RPE65	29579495	29574644	AS	•	•	NO
**c8.3(+)**	0	0	155	7.82E-42	••W	gg619	8	29604663	DEPDC1	29592933	29582597	AS	•	•	NO
**c12.1(+)**	0	0	61	1.27E-16	••W	gg1123	12	3516041	OGN	3642003	3652066	S	•	•	NO
**c12.1(+)**	0	0	61	1.27E-16	••W	gg1123	12	3516041	SHISA5	3493568	3482413	AS	•	•	NO
**c13.1(+)**	0	0	48	6.36E-13	••W	gg1005	13	6893210	GABRG2	6851434	6808843	AS	•	•	YES
**c13.1(+)**	0	0	48	6.36E-13	••W	gg1005	13	6893210	GABRA1	6985270	6945582	•	•	AS	YES

ERV Locus(Ori)	reads. H	reads L	reads W	P-value	Chicken	ERV Ref	Chr	Site	refGene	txStart	txEnd	ERV txUp	ERV txIn	ERV txDown	Sweep
**c18.1(+)**	0	0	46	1.36E-12	••W	gg619	18	524427	MYH1	456917	437938	AS	•	•	NO
**c18.1(+)**	0	0	46	1.36E-12	••W	gg619	18	524427	MYH3	499374	384631	AS	•	•	NO
**c18.1(+)**	0	0	46	1.36E-12	••W	gg619	18	524427	MYH2	500775	482485	AS	•	•	NO
**c18.1(+)**	0	0	46	1.36E-12	••W	gg619	18	524427	MYH6	570055	482579	•	AS	•	NO
**c18.1(+)**	0	0	46	1.36E-12	••W	gg619	18	524427	MYH1	611284	352393	•	AS	•	NO
**c18.1(+)**	0	0	46	1.36E-12	••W	gg619	18	524427	MYH6	611296	384713	•	AS	•	NO
**c18.1(+)**	0	0	46	1.36E-12	••W	gg619	18	524427	MYH3	611328	437920	•	AS	•	NO
**c18.1(+)**	0	0	46	1.36E-12	••W	gg619	18	524427	MYH3	632507	592807	•	•	AS	NO
**c20.1(+)**	35	0	0	4.07E-10	H••	gg9	20	11958	IGSF1	22576	25507	S	•	•	NO
**c20.1(+)**	35	0	0	4.07E-10	H••	gg9	20	11958	SFRS6	74296	69284	•	•	AS	NO
**c20.2(+)**	0	0	101	2.23E-27	••W	ALVA	20	10064772	ID1	9955624	9956740	•	•	S	NO
**c20.2(+)**	0	0	101	2.23E-27	••W	ALVA	20	10064772	TPX2	9989994	9999060	•	•	S	NO
**c20.2(+)**	0	0	101	2.23E-27	••W	ALVA	20	10064772	MYLK2	10005229	10013044	•	•	S	NO
**c20.2(+)**	0	0	101	2.23E-27	••W	ALVA	20	10064772	POFUT1	10099700	10104490	S	•	•	NO
**c20.2(+)**	0	0	101	2.23E-27	••W	ALVA	20	10064772	KIF3B	10104696	10113465	S	•	•	NO
**c20.2(+)**	0	0	101	2.23E-27	••W	ALVA	20	10064772	DNMT3B	10203705	10212614	S	•	•	NO
**c20.2(+)**	0	0	101	2.23E-27	••W	ALVA	20	10064772	MAPRE1	10214066	10222090	S	•	•	NO
**c20.2(+)**	0	0	101	2.23E-27	••W	ALVA	20	10064772	BCL2L1	9985476	9968507	AS	•	•	NO
**c20.2(+)**	0	0	101	2.23E-27	••W	ALVA	20	10064772	IRF10	10021470	10018362	AS	•	•	NO
**c21.1(+)**	0	0	144	5.08E-39	••W	gg428	21	5904693	NPPA	5763168	5765788	•	•	S	NO
**c21.1(+)**	0	0	144	5.08E-39	••W	gg428	21	5904693	MAD2L2	5847480	5851515	•	•	S	NO
**c21.1(+)**	0	0	144	5.08E-39	••W	gg428	21	5904693	C1orf187	5846629	5834625	AS	•	•	NO
**c23.1(+)**	0	76	0	6.12E-19	•L•	gg156	23	5763744	RPL11	5837002	5839342	S	•	•	YES
**c23.1(+)**	0	76	0	6.12E-19	•L•	gg156	23	5763744	TCEB3	5842344	5855501	S	•	•	YES
**c23.1(+)**	0	76	0	6.12E-19	•L•	gg156	23	5763744	LYPLA2	5859723	5863556	S	•	•	YES
**c23.1(+)**	0	76	0	6.12E-19	•L•	gg156	23	5763744	PNRC2	5884444	5888552	S	•	•	YES
**c23.1(+)**	0	76	0	6.12E-19	•L•	gg156	23	5763744	PPT1	5836864	5832209	•	•	AS	YES

ERV Locus(Ori)	reads. H	reads L	reads W	P-value	Chicken	ERV Ref	Chr	Site	refGene	txStart	txEnd	ERV txUp	ERV txIn	ERV txDown	Sweep
**c23.1(+)**	0	76	0	6.12E-19	•L•	gg156	23	5763744	SFRS13A	5899591	5891181	•	•	AS	YES
**c23.1(+)**	0	76	0	6.12E-19	•L•	gg156	23	5763744	FABP1	5904572	5902056	•	•	AS	YES
**c1.1(-)**	0	0	30	2.88E-08	••W	gg1093	1	25537606	KCND2	25579182	25310273	•	S	•	NO
**c1.2(-)**	0	0	63	5.68E-17	••W	gg377	1	25718392	KCND2	25579182	25310273	S	•	•	NO
**c1.3(-)**	0	0	35	1.52E-09	••W	gg1306	1	34362594	17.5	34420434	34444453	AS	•	•	NO
**c1.4(-)**	0	145	0	1.44E-36	•L•	gg448	1	48067508	NEDD1	47926746	47951589	•	•	AS	NO
**c1.4(-)**	0	145	0	1.44E-36	•L•	gg448	1	48067508	MIR135A-2	48192658	48192757	AS	•	•	NO
**c1.5(-)**	32	2	0	6.96E-08	HL•	gg1183	1	54697290	LARGE	54793760	54990691	AS	•	•	NO
**c1.6(-)**	86	0	0	8.32E-25	H••	gg1244	1	54862712	LARGE	54793760	54990691	•	AS	•	NO
**c1.7(-)**	27	0	0	2.42E-08	H••	gg1320	1	56567382	NFYB	56685114	56691879	AS	•	•	NO
**c1.7(-)**	27	0	0	2.42E-08	H••	gg1320	1	56567382	TXNRD1	56653900	56627717	•	•	S	NO
**c1.8(-)**	2	0	38	3.64E-09	H•W	gg105	1	64588199	AKR1B10	64618852	64610741	•	•	S	NO
**c1.9(-)**	0	0	189	2.38E-51	••W	gg370	1	65570918	MGST1	65427631	65434124	•	•	AS	NO
**c1.10(-)**	0	0	202	8.14E-55	••W	gg156	1	68099727	SOX5	68081128	68335941	•	AS	•	NO
**c1.11(-)**	31	41	0	3.03E-08	HL•	gg619	1	113736509	C21orf33	113827939	113834953	AS	•	•	NO
**c1.12(-)**	126	48	0	1.80E-24	HL•	gg619	1	135459454	GABRB3	135465533	135577048	AS	•	•	NO
**c1.13(-)**	60	95	0	4.68E-18	HL•	gg1088	1	166636687	TDRD3	166510209	166407687	S	•	•	NO
**c1.14(-)**	0	0	86	5.27E-23	••W	gg1167	1	169815642	PCDH8	169959398	169962915	AS	•	•	NO
**c1.14(-)**	0	0	86	5.27E-23	••W	gg1167	1	169815642	OLFM4	169859044	169834839	•	•	S	NO
**c2.1(-)**	0	0	157	8.88E-43	••W	gg1249	2	1415459	ADCYAP1R1	1268391	1369982	•	•	AS	NO
**c2.2(-)**	0	24	0	2.39E-06	•L•	gg1120	2	48148911	NT5C3	48041580	48053719	•	•	AS	NO
**c2.2(-)**	0	24	0	2.39E-06	•L•	gg1120	2	48148911	FKBP9	48074886	48059096	S	•	•	NO
**c2.3(-)**	0	15	180	1.77E-38	•LW	gg906	2	125628168	TPD52	125635083	125600210	•	S	•	NO
**c2.4(-)**	0	0	87	1.77E-23	••W	gg412	2	127375694	E2F5	127428844	127441377	AS	•	•	NO
**c2.5(-)**	0	34	45	1.94E-08	•LW	gg1244	2	143806500	FBXO32	143720333	143695679	S	•	•	YES
**c3.1(-)**	0	0	38	2.43E-10	••W	gg1212	3	1027426	FANCL	920584	891593	S	•	•	YES
**c3.2(-)**	0	0	30	2.88E-08	••W	gg1131	3	15998433	BMP2	16013577	16010636	•	•	S	NO

ERV Locus(Ori)	reads. H	reads L	reads W	P-value	Chicken	ERV Ref	Chr	Site	refGene	txStart	txEnd	ERV txUp	ERV txIn	ERV txDown	Sweep
**c3.3(-)**	0	0	32	9.02E-09	••W	gg1233	3	19385380	DUSP10	19447981	19470252	AS	•	•	NO
**c3.4(-)**	2	62	0	7.75E-14	HL•	gg1282	3	53808982	GTF2H5	53785643	53790123	•	•	AS	YES
**c3.5(-)**	1	0	92	9.68E-24	H•W	gg1320	3	56906046	MAP7	56993299	57101731	AS	•	•	NO
**c3.6(-)**	0	56	0	2.65E-14	•L•	gg382	3	70281167	OSTM1	70177176	70188136	•	•	AS	NO
**c3.6(-)**	0	56	0	2.65E-14	•L•	gg382	3	70281167	NR2E1	70146734	70136116	S	•	•	NO
**c3.7(-)**	0	0	42	2.55E-11	••W	gg1167	3	82770540	HTR1B	82820823	82821989	AS	•	•	NO
**c3.8(-)**	44	41	0	1.06E-09	HL•	gg1244	3	101711905	DDX1	101837290	101859293	AS	•	•	NO
**c4.1(-)**	0	52	0	4.92E-13	•L•	gg1131	4	13347887	DCX	13329700	13403236	•	AS	•	YES
**c4.2(-)**	0	116	12	1.43E-22	•LW	gg1245	4	16029904	GRIA3	16009119	15868384	S	•	•	NO
**c4.2(-)**	0	116	12	1.43E-22	•LW	gg1245	4	16029904	GRIA3	16009119	15868384	S	•	•	NO
**c4.3(-)**	82	169	0	2.78E-30	HL•	gg1249	4	53075646	CENPC1	53115873	53083976	•	•	S	NO
**c4.4(-)**	0	34	0	1.10E-08	•L•	ALV	4	59534816	ELOVL6	59493262	59560593	•	AS	•	NO
**c4.4(-)**	0	34	0	1.10E-08	•L•	ALV	4	59534816	EGF	59646098	59593970	•	•	S	NO
**c4.5(-)**	0	0	53	4.44E-14	••W	gg382	4	65806513	MIR383	65844694	65844767	AS	•	•	NO
**c4.6(-)**	14	69	0	1.31E-12	HL•	gg428	4	76755434	PPARGC1A	76629532	76695700	•	•	AS	NO
**c5.1(-)**	0	0	112	1.76E-30	••W	gg497	5	2738973	TMEM16E	2770252	2802690	AS	•	•	NO
**c5.1(-)**	0	0	112	1.76E-30	••W	gg497	5	2738973	SLC17A6	2823816	2853267	AS	•	•	NO
**c5.2(-)**	0	0	59	1.08E-15	••W	gg1157	5	43125662	TSHR	43202355	43250960	AS	•	•	YES
**c5.2(-)**	0	0	59	1.08E-15	••W	gg1157	5	43125662	DIO2	42994457	42977041	S	•	•	YES
**c5.3(-)**	5	66	0	1.02E-13	HL•	gg1093	5	50901807	EVL	50903042	50969097	AS	•	•	NO
**c5.3(-)**	5	66	0	1.02E-13	HL•	gg1093	5	50901807	YY1	50999597	51022607	AS	•	•	NO
**c5.4(-)**	0	91	0	5.34E-23	•L•	gg679	5	56076036	ERNI	56155476	56151622	•	•	S	YES
**c5.4(-)**	0	91	0	5.34E-23	•L•	gg679	5	56076036	ENS-1	56156062	56151615	•	•	S	YES
**c6.1(-)**	0	38	0	9.09E-10	•L•	gg153	6	18519457	NDUFB8	18495024	18497909	•	•	AS	NO
**c6.1(-)**	0	38	0	9.09E-10	•L•	gg153	6	18519457	SCD	18589084	18573465	•	•	S	NO
**c6.1(-)**	0	38	0	9.09E-10	•L•	gg153	6	18519457	CYP2C18	18664396	18655324	•	•	S	NO
**c6.2(-)**	0	0	182	3.13E-49	••W	gg462	6	19087088	RBP3	19079720	19065265	S	•	•	NO

ERV Locus(Ori)	reads. H	reads L	reads W	P-value	Chicken	ERV Ref	Chr	Site	refGene	txStart	txEnd	ERV txUp	ERV txIn	ERV txDown	Sweep
**c6.2(-)**	0	0	182	3.13E-49	••W	gg462	6	19087088	GDF2	19094749	19091528	•	•	S	NO
**c6.3(-)**	0	0	92	1.24E-24	••W	gg326	6	20550386	MIR107	20488044	20487964	S	•	•	NO
**c6.4(-)**	40	0	0	1.39E-11	H••	gg1280	6	33909591	KIAA0157	33884801	33907725	•	•	AS	NO
**c7.1(-)**	37	0	0	1.72E-10	H••	gg1282	7	26371197	CNTNAP5	26227278	25970551	S	•	•	NO
**c7.2(-)**	56	0	0	4.57E-16	H••	gg1318	7	29324935	UMPS	29451778	29457617	AS	•	•	NO
**c7.3(-)**	0	112	0	3.85E-28	•L•	gg377	7	37958636	PKP4	38002475	38069369	AS	•	•	NO
**c7.3(-)**	0	112	0	3.85E-28	•L•	gg377	7	37958636	ACVR1	37907942	37894082	S	•	•	NO
**c7.4(-)**	2	131	0	7.39E-31	HL•	gg462	7	38141077	PKP4	38002475	38069369	•	•	AS	NO
**c7.4(-)**	2	131	0	7.39E-31	HL•	gg462	7	38141077	WDSUB1	38186050	38161551	•	•	S	NO
**c7.4(-)**	2	131	0	7.39E-31	HL•	gg462	7	38141077	BAZ2B	38269262	38189619	•	•	S	NO
**c7.5(-)**	0	103	0	5.74E-26	•L•	gg497	7	38315122	WDSUB1	38186050	38161551	S	•	•	NO
**c7.5(-)**	0	103	0	5.74E-26	•L•	gg497	7	38315122	BAZ2B	38269262	38189619	S	•	•	NO
**c8.1(-)**	0	0	31	1.70E-08	••W	gg1294	8	1963660	MIR181A-1	2001560	2001664	AS	•	•	NO
**c8.1(-)**	0	0	31	1.70E-08	••W	gg1294	8	1963660	MIR181B-1	2001749	2001838	AS	•	•	NO
**c8.1(-)**	0	0	31	1.70E-08	••W	gg1294	8	1963660	PTPRC	2092242	2034797	•	•	S	NO
**c9.1(-)**	0	0	91	9.32E-25	••W	gg1123	9	1693770	DBR1	1588690	1580791	S	•	•	NO
**c9.2(-)**	0	0	88	6.08E-24	••W	gg1027	9	22139982	PDCD10	22157190	22168714	AS	•	•	NO
**c9.2(-)**	0	0	88	6.08E-24	••W	gg1027	9	22139982	SERPINI1	22156946	22117951	•	S	•	NO
**c9.3(-)**	0	28	0	1.24E-07	•L•	gg153	9	25368267	EIF2A	25227136	25214673	S	•	•	YES
**c9.3(-)**	0	28	0	1.24E-07	•L•	gg153	9	25368267	RNF13	25367113	25338075	S	•	•	YES
**c12.1(-)**	0	0	32	9.02E-09	••W	gg105	12	5327432	CNBP	5253628	5262898	•	•	AS	YES
**c12.1(-)**	0	0	32	9.02E-09	••W	gg105	12	5327432	RAF1	5183083	5155147	S	•	•	YES
**c12.1(-)**	0	0	32	9.02E-09	••W	gg105	12	5327432	ACAD9	5452360	5426113	•	•	S	YES
**c13.1(-)**	0	0	22	1.48E-06	••W	gg1183	13	9998020	DRD1	10106758	10105358	•	•	S	NO
**c15.1(-)**	0	0	52	3.35E-14	••W	ALVJ	15	7792842	PITPNB	7788016	7771362	S	•	•	NO
**c15.1(-)**	0	0	52	3.35E-14	••W	ALVJ	15	7792842	CHEK2	7934746	7922054	•	•	S	NO
**c17.1(-)**	0	0	107	1.01E-28	••W	gg428	17	2360348	GTF2H4	2231042	2269366	•	•	AS	NO

ERV Locus(Ori)	reads. H	reads L	reads W	P-value	Chicken	ERV Ref	Chr	Site	refGene	txStart	txEnd	ERV txUp	ERV txIn	ERV txDown	Sweep
**c17.1(-)**	0	0	107	1.01E-28	••W	gg428	17	2360348	COBRA1	2302413	2313548	•	•	AS	NO
**c17.1(-)**	0	0	107	1.01E-28	••W	gg428	17	2360348	ARRDC1	2361838	2388458	AS	•	•	NO
**c17.1(-)**	0	0	107	1.01E-28	••W	gg428	17	2360348	EHMT1	2391253	2490298	AS	•	•	NO
**c17.1(-)**	0	0	107	1.01E-28	••W	gg428	17	2360348	CACNA1B	2509604	2763633	AS	•	•	NO
**c17.1(-)**	0	0	107	1.01E-28	••W	gg428	17	2360348	TUBB2C	2279058	2276960	S	•	•	NO
**c20.1(-)**	0	42	0	9.76E-11	•L•	gg1054	20	12082236	CBLN4	12070717	12075259	•	•	AS	YES
**c20.1(-)**	0	42	0	9.76E-11	•L•	gg1054	20	12082236	C20orf43	11953582	11933109	S	•	•	YES
**c20.1(-)**	0	42	0	9.76E-11	•L•	gg1054	20	12082236	C20orf108	11993924	11990475	S	•	•	YES
**c24.1(-)**	0	0	22	1.48E-06	••W	gg1	24	1347156	BARX2	1366888	1393020	AS	•	•	YES
**c24.1(-)**	0	0	22	1.48E-06	••W	gg1	24	1347156	NFRKB	1465665	1463424	•	•	S	YES
**c27.1(-)**	2	57	0	5.23E-13	HL•	gg156	27	1274745	CCDC43	1210569	1202329	S	•	•	NO
**c27.1(-)**	2	57	0	5.23E-13	HL•	gg156	27	1274745	GJC1	1232266	1228979	S	•	•	NO
**c27.1(-)**	2	57	0	5.23E-13	HL•	gg156	27	1274745	EFTUD2	1259114	1243459	S	•	•	NO
**c27.1(-)**	2	57	0	5.23E-13	HL•	gg156	27	1274745	KIF18B	1279347	1272000	•	S	•	NO
**c27.1(-)**	2	57	0	5.23E-13	HL•	gg156	27	1274745	MAP3K14	1366543	1353335	•	•	S	NO
